# Efficacy of chloroquine, amodiaquine and sulphadoxine-pyrimethamine for the treatment of uncomplicated falciparum malaria: revisiting molecular markers in an area of emerging AQ and SP resistance in Mali

**DOI:** 10.1186/1475-2875-8-34

**Published:** 2009-02-26

**Authors:** Mamadou Tekete, Abdoulaye A Djimde, Abdoul H Beavogui, Hamma Maiga, Issaka Sagara, Bakary Fofana, Dinkorma Ouologuem, Souleymane Dama, Aminatou Kone, Demba Dembele, Mamadou Wele, Alassane Dicko, Ogobara K Doumbo

**Affiliations:** 1 Malaria Research and Training Center/Department of Epidemiology of Parasitic Diseases, Faculty of Medicine, Pharmacy and Odonto-Stomatology, Bamako, Mali

## Abstract

**Background:**

To update the National Malaria Control Programme of Mali on the efficacy of chloroquine, amodiaquine and sulphadoxine-pyrimethamine in the treatment of uncomplicated *falciparum *malaria.

**Methods:**

During the malaria transmission seasons of 2002 and 2003, 455 children – between six and 59 months of age, with uncomplicated malaria in Kolle, Mali, were randomly assigned to one of three treatment arms. *In vivo *outcomes were assessed using WHO standard protocols. Genotyping of *msp1*, *msp2 *and CA1 polymorphisms were used to distinguish reinfection from recrudescent parasites (molecular correction).

**Results:**

Day 28 adequate clinical and parasitological responses (ACPR) were 14.1%, 62.3% and 88.9% in 2002 and 18.2%, 60% and 85.2% in 2003 for chloroquine, amodiaquine and sulphadoxine-pyrimethamine, respectively. After molecular correction, ACPRs (cACPR) were 63.2%, 88.5% and 98.0% in 2002 and 75.5%, 85.2% and 96.6% in 2003 for CQ, AQ and SP, respectively. Amodiaquine was the most effective on fever. Amodiaquine therapy selected molecular markers for chloroquine resistance, while in the sulphadoxine-pyrimethamine arm the level of *dhfr *triple mutant and *dhfr*/*dhps *quadruple mutant increased from 31.5% and 3.8% in 2002 to 42.9% and 8.9% in 2003, respectively. No infection with *dhps *540E was found.

**Conclusion:**

In this study, treatment with sulphadoxine-pyrimethamine emerged as the most efficacious on uncomplicated falciparum malaria followed by amodiaquine. The study demonstrated that sulphadoxine-pyrimethamine and amodiaquine were appropriate partner drugs that could be associated with artemisinin derivatives in an artemisinin-based combination therapy.

## Background

All four human malaria species *Plasmodium falciparum, Plasmodium malariae, Plasmodium ovale *and *Plasmodium vivax *are present in Mali, but *P. falciparum *is responsible for 86.1 to 94.9% of all malarial infections [1-3]. During the peak transmission season, malaria is responsible for up to 25% of mortality in children, 40% of anaemia in pregnant women and 84% of hospital visits[3]. This major public health problem is worsened by the steady increase of the parasite's resistance to chloroquine (CQ), a cheap and safe anti-malarial drug [4,5]. The magnitude of anti-malarial drug resistance, its impact on morbidity and mortality in endemic countries and the scarcity of new anti-malarials have led some authors to warn for a malaria disaster in sub-Saharan Africa [5,6]. The continued usage of a failing drug does increase the rate of malaria mortality and morbidity, while a premature change of anti-malarial treatment policy may lead to an unsustainable economical burden on the health systems of endemic countries [7]. In order to promote evidence-based policy decisions, the World Health Organization (WHO) stressed the need to monitor the efficacy of anti-malarial drugs using standardized protocols [8]. This regular monitoring has led several National Malaria Control Programmes to switch their first line treatment from CQ to sulphadoxine-pyrimethamine (SP) or other anti-malarials [9]. However, resistance to SP emerged shortly afterwards in numerous places [10-12].

Amodiaquine (AQ) is a 4-amino quinoleine effective against chloroquine resistant (CQR) parasites, but seldom used because of a small risk of lethal toxicity [13]. However, a comprehensive review showed efficacy and safety data indicating the potential of AQ in areas where CQ has failed. Artemisinin derivatives are the most effective and rapid acting anti-malarials known to date. To deter resistance of malaria parasites to these drugs, their use is recommended in combination with a second anti-malarial with a longer half-life, a treatment regimen known as artemisinin-based combination therapy (ACT). For optimum efficacy of the combination, the artemisinin derivative ought to be combined with a drug that is efficacious in the area where it is to be used.

At the initiation of this study, the National Malaria Control Programme of Mali was in the process of formally changing its treatment guidelines to an ACT. Updated data on the efficacy of potential candidate monotherapies to be used in an ACT regimen in Mali are provided.

## Methods

### Study design

This was an open label clinical trial conducted in Kolle, Mali, during the malaria transmission seasons of 2002 (August 2002 – January 2003) and 2003 (July 2003 – January 2004). Kolle is a rural village of 2,500 people located 55 kilometers south of Bamako. Falciparum malaria is both endemic and seasonal with parasitaemia prevalence ranging from 40–50% in the dry season (October-May) and 70–85% in the rainy season (June-September) [14]. Inclusion criteria for this study included: 1) parental consent; 2) age between six and 59 months; 3) a positive malaria smear; 4) an axillary temperature between 37.5°C and 39.5°C; and 5) parasitaemia between 2,000 – 200,000 asexual parasites/μl. Exclusion criteria included: 1) haemoglobin < 5 g/dL; 2) clinical symptoms, such as prostration, respiratory distress, renal failure, hypoglycaemia, shock, bleeding, severe vomiting; and 3) a history of allergy or other severe adverse reaction to the study drugs. Children with any of the above exclusion characteristics or who were for any other reason determined to be too ill for inclusion in the study, or who declined to participate in the study, received standard and appropriate treatment, including parenteral quinine therapy, when indicated. Children were randomized into three treatment groups (CQ, AQ or SP). Treatments assignment was done by simple randomization, such that patients had a 2:1:1 chance to be assigned to the CQ, AQ and SP arms, respectively. Standard oral CQ and AQ were administered at 25 mg/kg over three days (10 mg/kg on days 0 and 1, 5 mg/kg on day 2). A single dose of 25 mg/kg of sulphadoxine and 1.25 mg/kg of pyrimethamine was administered on day 0. All subjects were observed for 60 minutes to monitor for adverse reactions and to make sure that the medicine was not vomited. If vomiting occurred within 30 mn, the full dose was re-administered. If vomiting occurred after 30 mn a half dose was re-administered. All acute concomitant illnesses were treated for free by the study team. Parental written informed consent was obtained before any protocol specific procedure was performed. The study was approved by the ethical committee of the Faculty of Medicine, Pharmacy and Odonto-Stomatology, Bamako, Mali.

### Data collection

Thick and thin malaria smears, venous blood, and finger prick blood-onto-filter paper samples were collected at the time of enrolment. Smears were Giemsa-stained. Filter papers were air-dried and stored for genetic analysis. With enrolment occurring on day 0, subjects were followed actively on days 1, 2, 3, 7, 14, 21 and 28 at the clinic and passively by 24-hour availability of a study clinician to evaluate and treat study subjects. At all active and passive follow-up times malaria smears and filter paper strips were obtained and brief history and physical evaluations made to seek signs or symptoms of persistent or recrudescent malaria. When cases of in vivo resistance were identified rescue treatment with oral or parenteral quinine was used. Cases of severe malaria in any subject evaluated at any time, including the first three days after treatment and whether or not they were enrolled in the study, were fully evaluated and admitted for appropriate medical care including parenteral quinine.

### Definition of in vivo resistance

In vivo data interpretation was done using WHO protocol for 14 and 28 days [8]. Treatment outcome were categorized as adequate clinical and parasitological response at day 14 (ACPR14) or at day 28 (ACPR28), early treatment failure (ETF14 and ETF28), late clinical failure (LCF14 and LCF28) and late parasitological failure (LPF14 and LPF28).

### Molecular analyses

#### DNA extraction

DNA was extracted from dried filter paper blood samples by the "methanol method" as described and five microliters of template were used for polymerase chain reaction (PCR) [15].

#### Nested mutation specific PCR for the detection of *pfcrt *K76T and pfmdr-1 polymorphisms (CQ and AQ arms)

Nested PCR followed by restriction endonuclease digestion was used to detect *pfcrt *K76T and *pfmdr-1 *polymorphisms, as described. Samples that failed to yield an unequivocal result by the restriction digestion method were re-analysed by the nested mutation specific PCR method, as previously described[15].

#### Molecular detection of *dhfr *and *dhps *mutations (SP arm)

Nested mutation-specific PCR and/or restriction digestion were performed according to published methods [16-18]. Because the *dhfr *mutations at codons 108, 51 and 59 [19] and the *dhps *mutations at codons 437 and 540 [20] have been suggested to be most associated with in vivo SP failure, the molecular analysis was focused on those mutations. Assays for all five mutations were performed on all in vivo resistant infections. Twice as many randomly selected pre-treatment infections were also included in the molecular analysis.

#### Molecular genotyping

DNA was obtained from day 0 and failure days samples. Initial DNA was extracted using the "methanol method"[15]. Samples that failed to yield interpretable results were re-extracted using a Qiagen Kit (Valencia, CA, USA) according to the manufacturer's instructions. Day 0 and failure days alleles of the merozoite surface proteins *msp-1*, *msp-2 *[21,22] and the microsatellite CA1 gene loci [23] were compared. A sample was classified as a true failure if it was recrudescent with all three markers. Cases that could not be resolved by this genotyping method were excluded from subsequent analyses. Molecular corrected ACPRs for day 14 and day 28 of follow up are hereafter designated as cACPR14 and cACPR28.

### Data analysis

Based on preliminary work in Mali the failure rate of CQ was estimated to 25%. For the comparator drugs (AQ and SP), the failure rate was estimated to 10% for each drug. Using an inverse sine transform approximation (Meinert, Cochran and Cox) and assuming an alpha error of 5% and a desired power of 90% with a desired sample size ratio of 2:1 (like double in CQ treatment group), 194 subjects in CQ treatment group and 98 subjects in each of AQ and SP treatment group were needed. In order to compensate for estimated 15% of lost-to-follow-up or -compliance data, a total of 456 subjects were expected (228 in CQ treatment group and 114 in each of AQ and SP treatment group) to be enrolled into the study.

Data gathered were double entered using Microsoft Access 2000 and analysed with STATA7 (stata corporation, Texas, USA). Bartlett test, Kruskal-Wallis, Chi Pearson and Fisher exact tests were used to compare variances, means and correlations. In all cases the significance was set at 5%.

## Results

**Figure 1:**
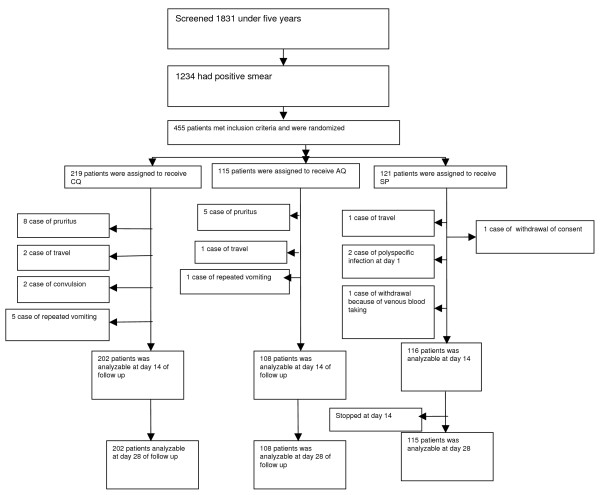
**Study profile**.

Of the 1,831 under five years of age patients screened, 1,234 had positive smears, and 455 subjects met our inclusion criteria (Figure 1). At baseline, the treatment arms were comparable with regard to mean age, sex, mean haemoglobin concentration and median parasitaemia (Table 1).

**Table 1:** Baseline characteristics of included children by treatment group

	CQ	AQ	SP	P
Mean age	3.10 (219)	2.85 (115)	2.92 (121)	NS
Sex (male)	49.77% (109)	47.83% (55)	50.41% (61)	NS
Fever	88.53% (193)	88.70% (102)	88.24% (105)	NS
Haemoglobin	9.75 (218)	9.85 (115)	9.86 (121)	NS
Median parasitaemia	30,075 (219)	24,525 (115)	24,000 (121)	NS

NS = Not significant

### Efficacy results

**Figure 2:**
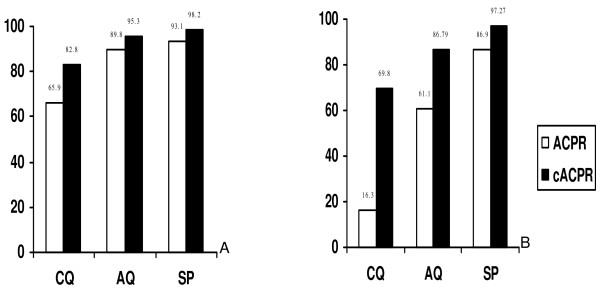
**ACPRs before and after molecular correction**. Panel A: Day 14 ACPRs; Panel B: Day 28 ACPRs.

Before PCR correction rates of ACPR14 and ACPR28 were unacceptably low for CQ (55.9% and 16.3%), moderate to high for AQ (89.8% and 61.1) and SP (93.1% and 86.9%), respectively (Table 2). However, the majority of the cases of failure were late clinical or parasitological failures. Only one case of ETF was observed with SP treatment (Table 2). After molecular correction, cACPR14 and cACPR28 remained bellow 90% for CQ and AQ but over 97% for SP (Figure 2).

**Table 2:** Clinical and parasitological response by treatment group

		ETF			LCF			LPF			ACPR*		
		CQ %(N)	AQ %(N)	SP %(N)	CQ %(N)	AQ %(N)	SP %(N)	CQ %(N)	AQ %(N)	SP %(N)	CQ %(N)	AQ %(N)	SP %(N)
		
14 Days	2002	5.4 (5)	0.0 (0)	0.0 (0)	7.6 (7)	0.0 (0)	0.0 (0)	38.0 (35)	5.7 (3)	7.3 (4)	48.9 (45)	94.3 (50)	92.7 (51)
	2003	7.3 (8)	5.5 (3)	1.6 (1)	3.6 (4)	1.8 (1)	0.0 (0)	27.3 (30)	7.3 (4)	4.9 (3)	61.8 (68)	85.5 (47)	93.4 (57)
	Total	6.4 (13)	2.8 (3)	0.9 (1)	5.4 (11)	0.9 (1)	0.0 (0)	32.2 (65)	6.5 (7)	6.0 (7)	55.9 (113)	89.8 (97)	93.1 (108)
	
28 Days	2002	5.4 (5)	0.0 (0)	0.0 (0)	20.7 (19)	1.9 (1)	0.0 (0)	59.8 (55)	35.8 (19)	11.1 (6)	14.1 (13)	62.3 (33)	88.9 (48)
	2003	7.3 (8)	5.5 (3)	1.6 (1)	20.0 (22)	5.5 (3)	0.0 (0)	54.5 (60)	29.1 (16)	13.1 (8)	18.2 (20)	60.0 (33)	85.2 (52)
	Total	6.4 (13)	2.8 (3)	0.9 (1)	20.3 (41)	3.7 (4)	0.0 (0)	56.9 (115)	32.4 (35)	12.2 (14)	16.3 (33)	61.1 (66)	86.9 (100)

*RCPA day 14: CQ *vs*. AQ, P < 0.001; CQ *vs*. SP, P < 0.001; AQ *vs*. SP, P = 0.3; RCPA day 28: CQ *vs*. AQ, P < 0.001; CQ *vs*. SP, P < 0.001; AQ *vs*. SP, P < 0.001

**Figure 3:**
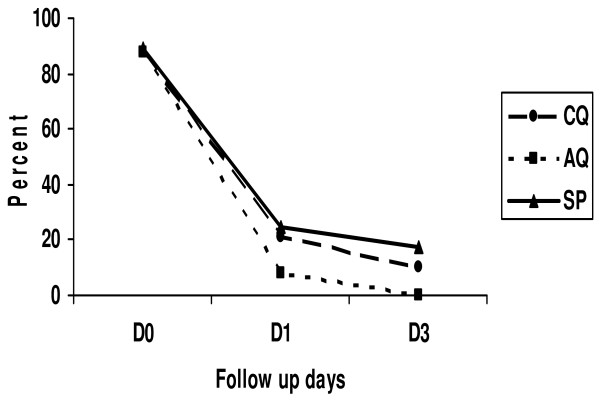
**Fever clearance by treatment arm**.

A net reduction of fever was seen between Day 0 and Day 2 in each of the treatment arms. At Day 2, there was a statistically significant difference between the CQ and SP arms for fever clearance (p < 0.05). No cases of fever were recorded in the AQ arm at Day 2, while 17.6% of children in the SP arm were febrile (Figure 3).

The level of anaemia in the whole population of the study was 51.5% (N = 454) in Kollé at baseline. By day 28 there was a non-significant decrease of anaemia prevalence to 42.9% (n = 163), 35.1% (n = 97) and 37.1% (n = 107) for CQ, AQ and SP, respectively. The level of pruritus was 3.7%, 3.5% and 0% in the CQ, AQ and SP arms, respectively.

### Prevalence of molecular markers of drug resistance

At baseline, 85.0% (n = 127) of the infections carried *pfcrt *76T and 49.3% (n = 136) had *pfmdr-1 *86Y in 2002. *Pfcrt *76T and *pfmdr-1 *86Y were present in 64.5% (n = 172) and 35.5% (n = 169), respectively in 2003. The *dhfr *108N, *dhfr *triple and *dhfr*/*dhps *quadruple mutation genotype were found in 45.5% (n = 55); 31.5% (n = 54) and 3.8% (n = 53), respectively, during the first year and 45.6% (n = 57), 42.9% (n = 56) and 8.9% (n = 56) during the second year. No case of *dhps *540E mutation was detected.

**Figure 4:**
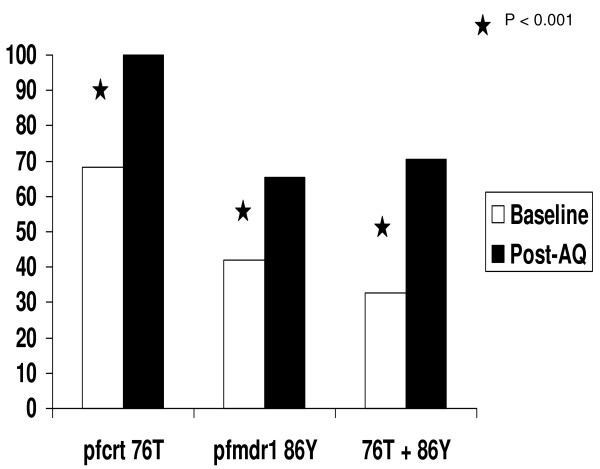
**Selection of molecular markers of CQ by AQ**.

The well-known selection effect of CQ treatment on *pfcrt *76T and *pfmdr-1 *86Y was also seen in this study. When these markers were considered separately, treatment failure in the AQ arm was neither associated with mutations in *pfcrt *(rates of *pfcrt *76T were 70%,(n = 10) in the failure group and 69% (n = 87) in the cured group; OR = 1.05, 95% CI = 0.22 – 5.60, p = 0.62) nor with *pfmdr-1 *(rates of *pfmdr-1 *86Y level were 63.6% (n = 11) in the failure group and 41.7% (n = 96) in the cured group, OR = 2.45, 95% CI = 0.59 – 10.80, p = 0.14). However, when the two mutations were considered together (*pfcrt *76T and *pfmdr-1 *86Y), there was a trend toward a significant association between the presence of both mutations in an infection and treatment failure with AQ (rate of *pfcrt *76T+*pfmdr-1 *86Y was 60% (n = 10) in the failure group and 32.2% (n = 87) in the cured group, OR = 3.16, 95% CI = 0.71–14.75, p = 0.08). Furthermore, each of these mutations was significantly selected in vivo by AQ monotherapy as was the combination of *pfcrt *76T and *pfmdr-1 *86Y (Figure 4).

## Discussion

At the initiation of this study, the National Malaria Control Programme of Mali needed fresh data on the level of resistance of Malian isolates of *P. falciparum *to CQ, AQ and SP. These data were required in order to choose the best partner drug to be combined with artemisinin derivates. The study showed that SP was the most efficacious drug (cACPR28 = 97.3%), followed by AQ (cACPR28 = 86.8%). The study also confirmed that CQ resistance levels had reached unacceptable levels (cACPR28 = 69.8%). Among the three treatment groups, AQ with 100% efficacy by day 2 was the most effective on fever, which is a key symptom of malaria. Because SP was largely used for intermittent preventive treatment in pregnancy (IPTp)[24] the Malian National Malaria Control Programme selected AS+AQ as first-line therapy, along with artemether-lumefantrine. These results are similar to several published reports, that also found SP to be superior to AQ and CQ [11,25,26].

The great majority of resistance cases found during this study were late parasitological failure. Indeed, even in the CQ arm where resistance was highest, only 6.4% of infections were early treatment failures. These rates are lower than resistance rates found in other parts of Africa [27,28] and confirm the observation that overall, anti-malarial resistance is lower in West Africa, compared to eastern and southern Africa [29].

Because AS/AQ is now one of the first line anti-malarial of Mali, the potential role of CQ resistant markers as potential determinants of AQ resistance was revisited. A non significant trend towards an association between the simultaneous presence of *pfcrt *76T + *pfmdr-1 *86Y and AQ treatment failure was found. This is consistent with previous similar reports from Nigeria and Burkina Faso [30-32]. Taken together, the available data suggests that the combined genotype *pfcrt *76T + *pfmdr-1 *86Y may be a useful molecular marker for AQ resistance in the field. This marker could be used as an interim tool for monitoring the efficacy of AS/AQ in the field as new molecular markers of artemisinin resistance are investigated. Indeed, a recent report showed that *pfcrt *and *pfmdr-1 *mutations were selected in vivo by AS+AQ treatment [33].

The prevalence of the *dhfr *triple mutant genotype rose from 31.5% to 42.9% and that of the *dhfr*/*dhps *quadruple mutant genotype evolved from 3.8% to 8.9% from 2002 to 2003, respectively but these differences were not statistically significant. No case of *dhfr *540E mutation was found during this study. Therefore, no quintuple mutation genotype was present indicating that SP resistance may be in its early days in this setting.

## Conclusion

SP was more efficacious than AQ, which in turn was better than CQ, for the treatment of uncomplicated falciparum malaria in Mali. Results of this study contributed to the change of anti-malarial treatment policy from CQ to ACT in Mali and to the choice of AQ as a partner drug for artemisinin. Although SP resistance was low, the rapid increase in the prevalence of molecular markers of SP resistance calls for a tight surveillance of the efficacy of this drug in the country.

## Competing interests

The authors declare that they have no competing interests.

## Authors' contributions

MT contributed to the study design, field studies, molecular analyses and drafted the manuscript. AD contributed to the design of the study, oversaw the field and laboratory studies, and to the writing and final approval of the manuscript. AHB, HM, BF, DO, SD, AK, DD and MW, conducted the field studies, collected the samples, performed the molecular analyses and assisted with manuscript preparation. IS and AD contributed to data analysis and manuscript writing. OKD contributed to study design and manuscript writing. All authors read and approved the final manuscript.
